# Co-Delivery of CPT-11 and Panobinostat with Anti-GD2 Antibody Conjugated Immunoliposomes for Targeted Combination Chemotherapy

**DOI:** 10.3390/cancers12113211

**Published:** 2020-10-31

**Authors:** Gils Jose, Yu-Jen Lu, Jung-Tung Hung, Alice L. Yu, Jyh-Ping Chen

**Affiliations:** 1 Department of Chemical and Materials Engineering, Chang Gung University, Kwei-San, Taoyuan 33302, Taiwan; D0523015@stmail.cgu.edu.tw; 2 Department of Neurosurgery, Chang Gung Memorial Hospital, Linkou, Kwei-San, Taoyuan 33305, Taiwan; luyj@cgmh.org.tw; 3 Institute of Stem Cell & Translational Cancer Research, Chang Gung Memorial Hospital, Linkou, Kwei-San, Taoyuan 33305, Taiwan; fllixhjt@cgmh.org.com (J.-T.H.); aiyu@health.ucsd.edu (A.L.Y.); 4 Department of Plastic and Reconstructive Surgery and Craniofacial Research Center, Chang Gung Memorial Hospital, Linkou, Kwei-San, Taoyuan 33305, Taiwan; 5 Research Center for Food and Cosmetic Safety, Research Center for Chinese Herbal Medicine, College of Human Ecology, Chang Gung University of Science and Technology, Taoyuan 33305, Taiwan; 6 Department of Materials Engineering, Ming Chi University of Technology, Tai-Shan, New Taipei City 24301, Taiwan

**Keywords:** liposome, targeted delivery, antibody, cancer therapy, drug delivery system, CPT-11, panobinostat, nanomedicine

## Abstract

**Simple Summary:**

In targeted cancer therapies, liposomes conjugated with antibody (Ab) can selectively deliver drugs to antigen-expressing cancer cells through active targeting and improve anti-cancer efficacy. Many glioblastoma cell lines and primary biopsies express high levels of disialoganglioside GD2 antigen, making it an excellent candidate for targeted cancer therapy. In this study, we prepared anti-GD2 Ab conjugated immunoliposomes (ImmuLipCP) for co-delivery of CPT-11 and panobinostat, which is intended for combination targeted chemotherapy. To compare the GD2 targeting mechanism, we used glioma cells with low GD2 expression (U87MG) and its drug-resistant variant with high GD2 expression (U87 DR). We demonstrate that ImmuLipCP show enhanced cytotoxicity against U87DR through Ab-mediated intracellular trafficking and drug delivery, for synergistic cancer cell killing effects. Using a xenograft tumor model by subcutaneous implantation of U87DR in nude mice, we also validate the targeting and anti-cancer efficacy of ImmuLipCP in vivo.

**Abstract:**

The consistent expression of disialoganglioside GD2 in neuroblastoma tumor cells and its restricted expression in normal tissues open the possibility to use it for molecularly targeted neuroblastoma therapy. On the other hand, immunoliposomes combining antibody-mediated tumor recognition with liposomal delivery of chemotherapeutics have been proved to enhance therapeutic efficacy in brain tumors. Therefore, we develop immunoliposomes (ImmuLipCP) conjugated with anti-GD2 antibody, for targeted co-delivery of CPT-11 and panobinostat in this study. U87MG human glioma cell line and its drug resistant variant (U87DR), which were confirmed to be associated with low and high expression of cell surface GD2, were employed to compare the targeting efficacy. From in vitro cytotoxicity assay, CPT-11 showed synergism drug interaction with panobinostat to support co-delivery of both drugs with ImmuLipCP for targeted synergistic combination chemotherapy. The molecular targeting mechanism was elucidated from intracellular uptake efficacy by confocal microscopy and flow cytometry analysis, where 6-fold increase in liposome and 1.8-fold increase in drug uptake efficiency was found using targeted liposomes. This enhanced intracellular trafficking for drug delivery endows ImmuLipCP with pronounced cytotoxicity toward U87DR cells in vitro, with 1.6-fold increase of apoptosis rate. Using xenograft nude mice model with subcutaneously implanted U87DR cells, we observe similar biodistribution profile but 5.1 times higher accumulation rate of ImmuLip from in vivo imaging system (IVIS) observation of Cy5.5-labelled liposomes. Taking advantage of this highly efficient GD-2 targeting, ImmuLipCP was demonstrated to be an effective cancer treatment modality to significantly enhance the anti-cancer therapeutic efficacy in U87DR tumors, shown from the significant reduced tumor size in and prolonged survival time of experiment animals as well as diminished expression of cell proliferation and enhanced expression of apoptosis marker proteins in tumor section.

## 1. Introduction

Malignant gliomas, the most common and aggressive subtype of primary brain tumor, are receiving considerable attention from physicians and researchers mainly because of their incurable nature [1]. Although various therapeutic inventions are increasingly emerging, the recurrence of malignant cells within a few months has enabled the definition of such tumor as the most difficult neoplasms to treat [2]. The highly proliferative and infiltrative nature of brain glioma cells and its resistive nature towards many chemotherapeutic agents further enhances the complexity of glioma treatment [3]. Even though various chemotherapy drugs are available for clinical applications, the poor capability of these drugs to penetrate the blood brain barrier and to attain desired doses of systemic chemotherapeutics demands the development of novel drug delivery system for effective glioma therapy [4].

Nano-sized drug carriers made of various materials, such as polymers, metal particles, lipids and so forth, have entered into the pharmaceutical market with the aim of enhancing the therapeutic properties of various drugs [5,6]. Due to the characteristic features of nanocarriers to store the drugs within themselves and to release the desirable amount of drug in the right place, nanoparticle-based drug delivery system (DDS) shows higher potentials in overcoming drawbacks of current chemotherapy drugs, including unwanted side effects due to non-specificity and low availability [7,8]. Owing to the unique characteristics such as biodegradability, biocompatibility and amphiphilic nature, liposomes have shown enormous potential as a drug carrier for cancer therapy [9]. Liposomes are unilamellar or multilamellar vesicles with interior aqueous space, composed of self-assembled lipid molecules and can carry both hydrophobic and hydrophilic dugs [10,11]. As liposomes can act as a long circulating nanocarriers, drug-loaded liposomes can lead to site-specific accumulation of drugs [12]. Moreover, liposomes may provide a consistent level exposure of the targeted tissue to the drug, due to its sustained release characteristics [11]. So far various formulations of liposomes, containing both hydrophilic and hydrophobic chemotherapeutics, are available for research and clinical studies [13]. Among these, CPT-11 (irinotecan)-loaded liposomes has received tremendous attention for the treatment of various cancers through the inhibition of the DNA eukaryotic enzyme topoisomerase I [14].

The CPT-11, 7-ethyl-10-[4-(1-piperidino)-1-piperidino]-carbonyloxy-camptothecin, has long been known for its auspicious role in the treatment of various cancers [15]. As a topoisomerase I inhibitor, CPT-11 is capable to inhibit DNA replication and thereby exerts cytotoxicity towards cancer cells [16,17]. When irinotecan comes in contact with glioma cells, it undergoes carboxylesterase mediated breakdown and produces the metabolite called SN-38, which is known to be 100–1000 times more potent as an inhibitor of topoisomerase [18,19]. Although CPT-11 is capable of crossing the blood brain barrier, studies have shown that the response rate attained from CPT-11 single drug chemotherapy was in between 0 and 44% with a survival rate of 2 to 11 months [20]. Therefore, combination therapy using CPT-11 with some other drugs was introduced and shown to offer a better option for enhancing the chemotherapeutic efficacy [21]. Panobinostat (Farydak^®^) is a Food and Drug Administration (FDA) approved histone deacetylase inhibitor for multiple myeloma treatment [22]. As a potent inhibitor of histone deacetylase, panobinostat prevents the deacetylation of histone and nonhistone proteins and results in cellular differentiation, cell-cycle progression and regulation of gene transcription and apoptosis [23]. Chemically, this deacetylase (DAC) inhibitor is capable to induce apoptosis in multiple myeloma cells even at low concentrations. Compared to other DAC inhibitors, panobinostat leads to elongated hyperacetylation of the histone protein and prominent alterations of gene expressions in tumor cells in contrast to healthy cells [24].

The anticancer properties of the commercially available drugs were well explored in combination with nanotechnology [25]. Due to the introduction of nanoparticle-based drug delivery system (DDS), we were able to overcome the drawbacks of most of the chemotherapeutics including non-specific targeting and related drug toxicity, which is being considered as the main obstacle of conventional chemotherapy [26]. Although nanocarrier-based DDS offers many advantages over conventional chemotherapy, poor accessibility of antineoplastic agents to the tumor results in lower drug dosage and the resultant transient responses [27]. Hence, targeting tumors by means of active targeting, by conjugating the nanocarriers with a ligand that can specifically bind to the receptor expressed on tumor cell surface, could be envisioned as a viable means to enhance the cancer treatment efficiency [28]. Toward this end, a number of targeting ligands were investigated for the development of targeted liposomes in drug delivery, including peptides, carbohydrates and antibodies [29]. Among them, antibody-conjugated liposomes (immunoliposomes), that can target antigen-expressing cancer cells, have attracted considerable attention for targeted cancer therapies as they can selectively deliver drugs to tumor cells with improved anti-cancer efficacy and reduced toxicity [30].

The GD2 is a disialoganglioside that is consistently expressed on tumors of neuroectodermal origin such as human neuroblastoma. Besides neuroblastoma, several neuroectoderm-derived neoplasms, including glioblastoma and sarcomas express high levels of the tumor-associated antigen GD2 [31]. Similar to several pediatric and adult solid tumors, a variety of glioblastoma cell lines and primary biopsies commonly express high levels of GD2 antigen [32]. Due to its high density on tumor cells as well as its restricted expression on normal tissue, GD2 is an excellent candidate for targeted cancer therapy [33,34]. As a matter of fact, the National Cancer Institute has ranked GD2 as the 12th most important cancer antigen from its pilot program for prioritization of cancer antigens [35]. Specifically, immunoliposomes conjugated with whole or fragment of anti-GD2 antibody (Ab) have been explored for targeted delivery of doxorubicin [34,36], antisense oligodeoxynucleotides [37], fenretinide [38] and survivin inhibitor [39] to tumor cells. 

**Figure 1:**
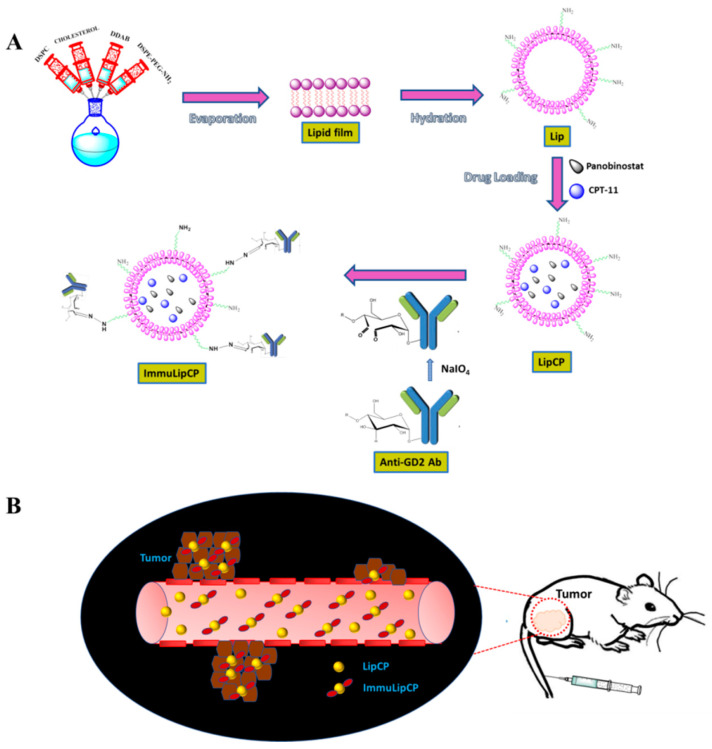
(**A**) The liposomes (Lip) were formed from 1,2-distearoyl-sn-glycero-3-phosphocholine (DSPC), dimethyldioctadecylammonium bromide (DDAB), cholesterol and N-(aminopropyl polyethyleneglycol)carbamyl-distearoylphosphatidyl-ethanolamine (DSPE-PEG-NH_2_) using the thin film hydration method. The drugs were loaded into liposomes by repeated freeze-thaw cycles to prepare CPT-11 and panobinostat-loaded liposomes (LipCP). The LipCP was surface conjugated with anti-GD2 Ab by forming covalent bonds between surface amine groups in liposomes and aldehyde groups in activated anti-GD2 Ab that is formed after NaIO_4_-catalyzed ring opening of the sugar moiety in the Ab. (**B**) The ImmuLipCP could show enhanced cytotoxicity toward tumor cells overexpressing GD2 over LipCP.

In this study, we prepared anti-GD2 Ab conjugated immunoliposomes (ImmuLipCP) for co-delivery of CPT-11 and panobinostat (Figure 1). We hypothesized that ImmuLipCP will show enhanced cytotoxicity against tumor cells overexpressing GD2, through anti GD2 Ab-mediated intracellular trafficking and drug delivery, for synergistic cancer cell killing effects (Figure 1). This hypothesis will be tested by comparing the targeting efficiency of liposomes without anti-GD2 Ab (Lip) or liposomes modified with low (ImmuLip-L) or high (ImmuLip) density of anti-GD2 Ab. To elucidate the GD2 targeting mechanism, we explored the use of glioma cells with low GD2 (U87MG) and high GD2 expression (U87 DR, a drug-resistant cell line derived from U87MG) in this study. Finally, we conduct a proof-of-concept study using a xenograft tumor model by subcutaneous implantation of U87DR in nude mice to validate the targeting and anti-cancer efficacy of ImmuLipCP.

## 2. Results and Discussion

### 2.1. Physico-Chemical Characterization of Liposomes

We prepare blank liposomes in this study by the thin-film hydration method, followed by drug loading through repeated freeze-thaw cycles (Figure 1A). After conjugation of anti-GD2 Ab to liposomes through spontaneous covalent bonds formed between aldehyde groups in activated Ab and amine groups on liposome surface, we obtain the drug-loaded immunoliposome (ImmuLipCP) (Figure 1A). Using 4 mg anti-GD2 Ab for conjugation with 5 mg Lip, 65% Ab conjugation efficiency was found for the Ab, which translates to 0.52 mg GD2/mg liposome. The average particle size from dynamic light scattering (DLS) as well as zeta potential were determined for various liposomal formulation. As shown in Table 1, the particle size of all prepared liposomes was below 200 nm, with significant increase of hydrodynamic diameter originated from Ab conjugation to liposome surface with the high molecular weight of immunoglobulins. Nonetheless, loading of drugs within the aqueous core of liposome did not lead to significant change of particle size as expected. The polydispersity index (PDI) is below 0.30 for all preparations, indicating a homogenous distribution of the lipid vesicles as well as a stable suspension of liposomes [40]. The average zeta potentials are 13.3 and 14.7 mV and show no significant difference between Lip and LipCP from electrophoretic mobility measurements due to the presence of cationic lipid dimethyldioctadecylammonium bromide (DDAB) in the lipid bilayer (Table 1). The zeta potential also shows no significant difference between blank and drug-loaded immunoliposomes (ImmuLip vs ImmuLipCP). Nonetheless, the zeta potential decreased (9.6 and 8.3 mV) after conjugation with ligand, arising from the slightly net negative charge associated with anti-GD2 Ab (isoelectric point = 7.3) and the consumption of the amine groups on liposome surface during the conjugation step.

**Figure 2:**
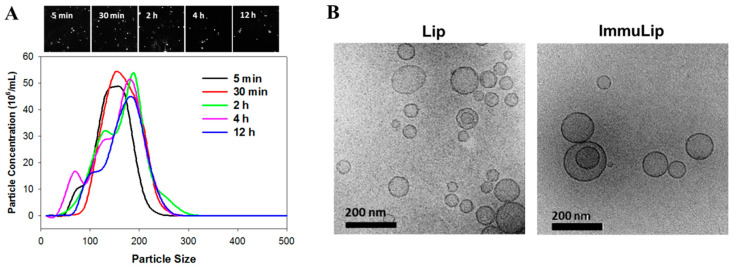
(**A**) The stability of CPT-11 and panobinostat-loaded immunoliposomes (ImmuLipCP) in 10% fetal bovine serum (FBS)/90% phosphate buffered saline (PBS) solution as determined by nanoparticle tracking analysis (NTA) with the screenshot images showing the light scattering particles. (**B**) The morphology of liposomes (Lip) and immunoliposomes (ImmuLip) from cryo-transmission electron microscopy (cryo-TEM) analysis (Bar = 200 nm).

To determine the in vivo-relevant colloidal stability of drug-loaded immunoliposomes, the particle size of ImmuLipCP was determined in 10% fetal bovine serum (FBS) diluted in 90%phosphate buffered saline (PBS) at different time points using nanoparticle tracking analysis (NTA). As shown in Figure 2A, the peak particle size of liposome apparently increased with incubation time. However, decrease of peak particle concentration was noted with simultaneous appearance of some smaller diameter liposome population, possibly due to destruction of liposomes. Nonetheless, no liposome with size above 300 nm was detected even after 12 h incubation. The stability of liposomes depends on many factors and particle aggregation may lead to increase in size and decrease of counts from NTA analysis [41]. Overall, the stability of ImmuLipCP determined from NTA endorses their capability for targeted drug delivery and intracellular uptake by cancer cells. We further determined the morphology of liposomes by observation with a cryo-transmission electron microscope (cryo-TEM). As shown in Figure 2B, the morphology of liposomes (Lip) and immunoliposomes (ImmuLip) were spherical in shape with an aqueous core enclosed by lipid bilayer. The particle size in the range of 50 to 200 nm, which is consistent with the hydrodynamic particle diameter measured from DLS analysis. Most liposomes are unilamellar vesicles as shown from the cryo-TEM image with minimum multilamellar vesicles after the extrusion process [42]. With higher internal aqueous volume, the unilamellar liposomes may be more efficient in encapsulating hydrophilic drugs such CPT-11 and panobinostat than multilamellar liposomes.

### 2.2. Drug Loading and Release

**Figure 3:**
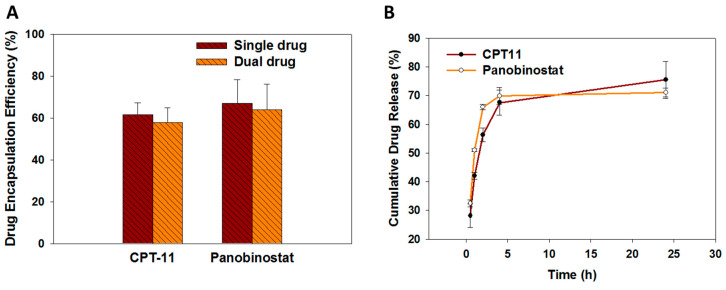
(**A**) The drug encapsulation efficiency of CPT-11 (single drug), panobinostat (single drug) or CPT-11 + panobinostat (dual drug) in immunoliposomes (*n* = 5). (**B**) The drug release profiles from CPT-11 and panobinostat-loaded immunoliposomes (ImmuLipCP) at 37 °C in PBS (pH 7.4) (*n* = 5).

The encapsulation efficiency (EE) of drugs into immunoliposomes was studied based on pilot experiments conducted to optimize the ratio between CPT-11 and panobinostat. Using 2 mg/mL CPT-11 and 0.5 mg/mL panobinostat for drug loading, we achieved the optimum EE of both drugs, which is 57.8 ± 7.2% (CPT-11) and 63.7 ± 12.3% (panobinostat) (Figure 3A). These values could be compared with the EE when each drug was encapsulated separately in the liposomes, which are 61.5 ± 5.8% and 66.8 ± 11.4% for CPT-11 and panobinostat, respectively. As no significant difference was found between the EE when a drug was loaded either alone or in the presence of the other drug, we conclude that minimum interference between drugs exists during drug loading, which might hamper the EE during co-encapsulation of both drugs. This underlines the feasibility to co-encapsulate both drugs within the aqueous core of ImmuLipCP for co-delivery of CPT-11 and panobinostat. The final preparation of ImmuLipCP with 0.06 mg CPT-11/mg liposome and 0.26 mg panobinostat/mg liposome were used for following studies. We postulate that ImmuLipCP could show synergistic cancer cell killing effects by co-delivery of CPT-11 and panobinostat at this drug concentration.

The release profiles of CPT-11 and panobinostat from ImmuLipCP were evaluated in phosphate buffered saline (pH 7.4) at 37 °C. As shown in Figure 3B, both drugs exhibit a biphasic drug release profile that was consistent with drug release from liposomes [43]. A burst release of each drug from liposomes was observed up to 5 h with faster release rate of panobinostat than CPT-11. The release percentages of both drugs reached 70–75% after 24 h with no significant difference found between them.

### 2.3. In Vitro Experiments

#### 2.3.1. Glioma Cell Lines with Different GD2 Expression and Intracellular Uptake of Immunoliposomes 

In order to study the effect of GD2 expression level on targeting efficacy of ImmuLip, we first develop palbociclib-resistant glioma cells (U87DR) with higher GD2 expression than U87MG in this study. Using a gradient of palbociclib concentration from 0.2 µM to 1 μM, we observed morphological change of U87MG to its variant U87DR within 3 months (Supplementary Figure S1). The exact nature of GD2 expression was determined from flow cytometry analysis, which gave 16.1% and 43.9% GD2-positive cells and 136 and 542 mean fluorescence intensity for U87MG and U87DR cell lines, respectively. We thus achieve the goal of obtaining a high GD2 expressing glioma cell line by using a drug-resistant variant of U87MG after screening with palbociclib. The four times higher expression of GD2 in U87DR glioma cells justify its use for following in vitro and in vivo studies to elucidate the effect of anti GD2 Ab-mediated targeted drug delivery by ImmuLipCP.

**Figure 4:**
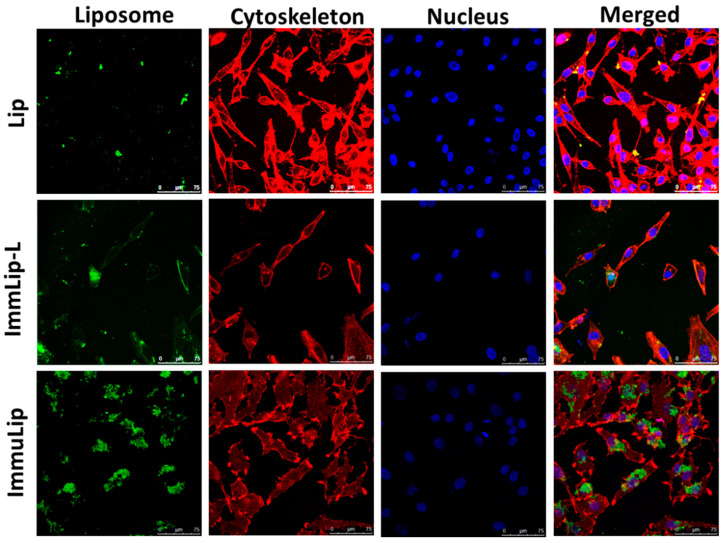
The intracellular uptake of liposomes (Lip), immunoliposomes (ImmuLip) and immunoliposomes prepared with 1/4 of anti-GD2 Ab used for ImmuLip (ImmuLip-L) by U87DR cells in 24 h from confocal laser scanning microscopy analysis (bar = 75 μm). Liposomes were labelled with 5(6)-carboxyfluorescein N-hydroxysuccinimide ester (green), cytoskeleton labelled with phalloidin-tetramethylrhodamine B isothiocyanate (red) and nuclei counterstained with Hoechst (blue).

Intracellular uptake of liposomes fluorescently labelled with 5(6)-carboxyfluorescein N-hydroxysuccinimide ester (fluorescein NHS) was first evaluated qualitatively by incubating U87DR cells separately with Lip or ImmuLip for 24 h (Figure 4). The uptake efficiency of green fluorescence-producing liposomes was examined using a confocal laser scanning microscope after staining the cell cytoskeleton with red fluorescence-producing phalloidin-tetramethylrhodamine B isothiocyanate and the nucleus with blue fluorescence-producing Hoechst 33342. To elucidate the effect of ligand density on cellular trafficking, a different immunoliposome (ImmuLip-L) was prepared by following the same procedure as ImmuLip but by reacting 1 mg anti-GD2 Ab (1/4 of ImmuLip) with 5 mg Lip. The ImmuLip was prepared with 61% Ab conjugation efficiency and 0.12 mg GD2/mg liposome. As shown in Figure 4, the fluorescence intensity obtained for ImmuLip was significantly stronger in comparison to both Lip and ImmuLip-L. It is clear from the confocal image that, the internalization efficiency of liposomes by U87DR, as revealed form the green fluorescence signal area associated with liposome, is in the order of ImmuLip >> ImmuLip-L > Lip. As the difference in intracellular uptake is correlated with the presence and density of ligand, we ascribe this effect to be originated from anti-GD2 Ab conjugated to the liposome surface. Taken together, our study underlines the importance of ligand density on antigen-Ab mediated intracellular trafficking and endorse the preference of using ImmuLip for targeted drug delivery.

**Figure 5:**
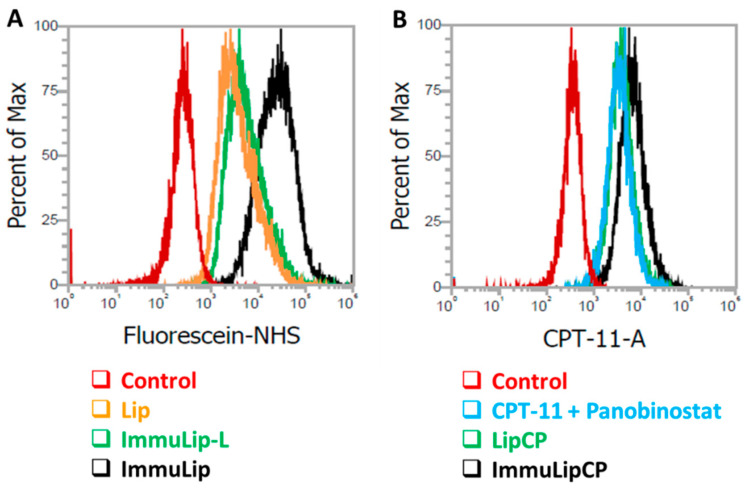
(**A**) The intracellular liposome uptake efficiency from flow cytometry analysis after incubating U87DR cells with fluorescein-labelled Lip, ImmuLip-L and ImmuLip for 24 h. (**B**) The intracellular drug uptake efficiency of from flow cytometry analysis of CPT-11 blue fluorescence after incubating U87DR cells with free drugs (CPT-11 + panobinostat), LipCP and ImmuLipCP for 24 h.

The intracellular uptake efficiency of different liposomes into U87DR was further subject to quantitatively analysis using flow cytometry, which can determine the intracellular fluorescence signal intensity associated with fluorescein-labelled liposomes. As shown from Figure 5A, the geometric mean fluorescence intensities after 24 h incubation with U87DR from fluorescence-activated cell sorting (FACS) analysis are 5945, 8743 and 35,918 for Lip, ImmuLip-L and ImmuLip, respectively. In line with the trends observed from confocal microscopy analysis (Figure 4), ImmuLip is shown with 6 times higher targeting efficacy than Lip through the action of abundant anti-GD2 Ab on liposome surface for mediating cellular trafficking. 

As cytotoxicity toward cancer cells is related to the intracellular drug concentration, the effectiveness of ImmuLipCP as a DDS was further examined from flow cytometry analysis. This was accomplished by taking advantage of the blue fluorescence signal associated with CPT-11 when U87DR cells were treated with free drugs, LipCP or ImmuLipCP for 24 h (Figure 5B). As in particle uptake, concomitant increase of intracellular CPT-11 fluorescence intensity was found for ImmuLipCP vs. LipCP, with the geometric mean intensities being 7294 and 4255, respectively. That similar drug uptake efficiency with merged peaks in Figure 5B found between free drugs (CPT-11 + panobinostat) (geometric mean intensity = 4064) and LipCP indicates drug delivery with non-targeted liposomal formulation could not improve the passive delivery of drugs into cancer cells. This further underlines the importance of targeted drug delivery by using anti-GD2 Ab as a ligand to enhance the cytotoxicity toward GD2 overexpressing U87DR. It should be noted the 1.8-fold increase in the drug uptake efficacy using ImmuLip over Lip is lower than the 6-fold increase shown in particle uptake efficacy. The difference between particle and drug uptake efficiency may be due to premature drug release before intracellular uptake of liposomes. Nonetheless, ImmuLip could be deemed as an efficient DDS for targeted drug delivery to enhance cytotoxicity toward GD2-overexpressing U87DR cell line. 

#### 2.3.2. In Vitro Cytotoxicity of Free Drugs

**Figure 6:**
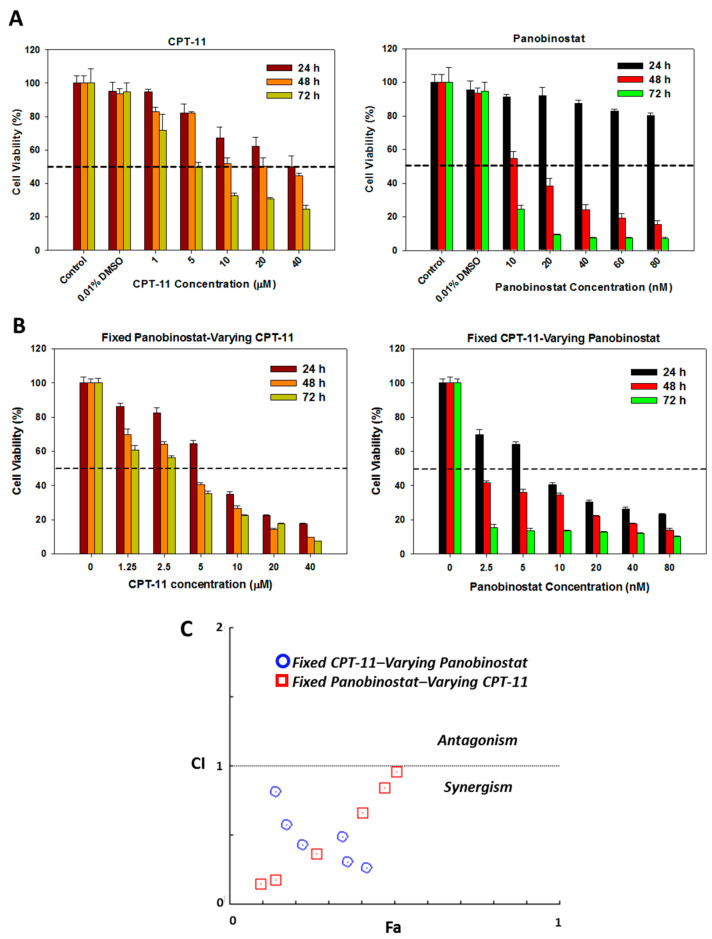
The cytotoxicity of single drug (CPT-11 or panobinostat) (**A**) and dual drug (CPT-11 and panobinostat) (**B**) toward U87DR at different time points (*n* = 5). (**C**) The synergistic effect of drug combination was analyzed by CompuSyn software with results taken from (**B**), from which the combination index (CI) value could be used to categorize the drug interaction into synergism (CI < 1), additive (CI = 1) and antagonism (CI > 1).

To support co-delivery of CPT-11 and panobinostat using liposomes, we determine the synergism of the drug combination toward U87DR cells with different concentrations of CPT-11 and/or panobinostat. The cytotoxicity was determined from relative cell viability using (3-(4,5-dimethylthiazol-2-yl)-5-(3-carboxymethoxyphenyl)-2-(4-sulfophenyl)-2H-tetrazolium) (MTS) assays, which is a calorimetric method for the sensitive quantification of viable cells when NAD(P)H-dependent dehydrogenase enzymes present in viable cells converting the MTS tetrazolium compound to a soluble and colored formazan product [44]. As shown in Figure 6A, the cytotoxicity of CPT-11 increases as the concentration of the drug increases from 1 µM to 40 µM. In comparison to 24 h incubation, the cytotoxicity shows an increasing trend when cells were incubated with the drug for longer durations at 48 h and 72 h. The IC_50_ values were in the order of 40 µM, 20 µM and 5 µM for 24 h, 48 h and 72 h respectively. Similarly, panobinostat also shows an increase in cytotoxicity when its concentration increases from 10 nM to 80 nM (Figure 6A). Although the cytotoxicity of panobinostat was not predominant after 24 h incubation within the tested concentrations, a rapid decrease of cell viability was noted at longer incubation duration (48 h and 72 h). 

Combination of panobinostat and CPT-11 at different ratios leads to further decrease in cell viability (Figure 6B). As expected, the relative cell viability using dual drug by either fixing panobinostat concentration (20 nM panobinostat) or fixing CPT-11 concentration (2 µM) shows drastic decline when compared to those obtained by using single drug. Most importantly, the dual drug cytotoxicity data offer the possibility to determine the synergistic effect of drug combination, as analyzed from the CompuSyn software with the non-constant ratio combination method (Figure 6C). This software was used to calculate the combination index (CI) value from results in Figure 6B, based on which the drug interaction could be categorized into synergism (CI < 1), additive (CI = 1) or antagonism (CI > 1) effect [45]. As shown in Figure 6C, the CI values obtained for all combinations of panobinostat and CPT-11 are between zero and one, thereby confirming the combination of these two drugs will lead to drug synergism for synergistic cancer cell killing effect toward U87DR. It also supports the rational to use ImmuLipCP for co-delivery of CPT-11 and panobinostat in synergistic cancer chemotherapy.

#### 2.3.3. Biocompatibility of Liposomes and Apoptosis Induced by Drug-Loaded Liposomes

**Figure 7:**
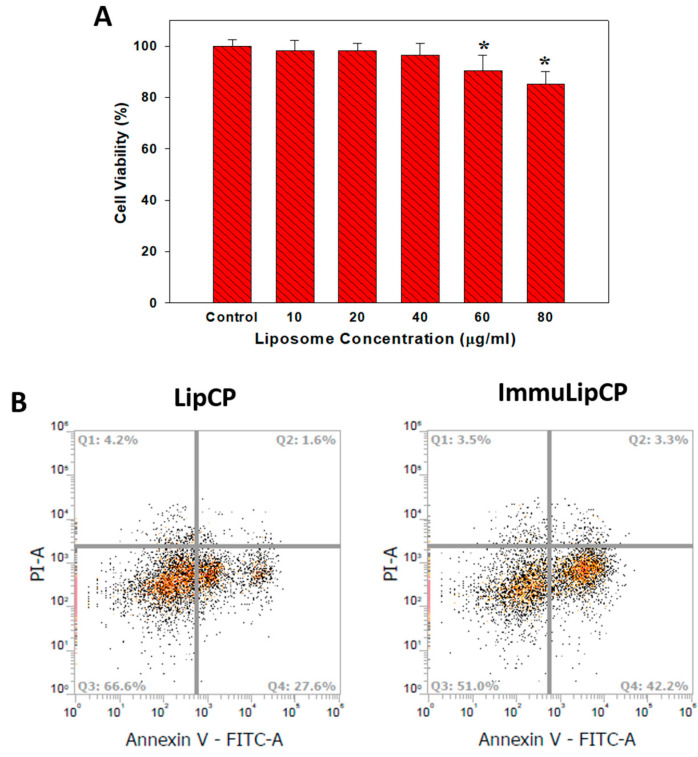
(**A**) Biocompatibility of ImmuLip by determining the relative cell viability using MTS assays when U87DR cells were treated with different concentrations of liposome for 24 h (*n* = 5). * *p* < 0.05 compared with control. (**B**) The flow cytometry analysis for cell apoptosis after 24 h incubation of U87DR with drug-loaded non-targeted liposomes (LipCP) and targeted liposomes (ImmuLipCP) at 37 °C. The cells were stained with Annexin V/PI and apoptotic and necrotic cells were visualized (Q1: necrotic; Q2: late apoptotic; Q3: live; Q4: early apoptotic). The concentration of panobinostat and CPT-11 were fixed as 5 nM and 2 μM for each experiment group.

To study the apoptosis induced by drug-loaded liposomes, we first confirm the biocompatibility of ImmuLip by determining the relative cell viability using MTS assays when U87DR were treated with different concentrations of liposome for 24 h. As shown in Figure 7A, there is no significant difference in cell viability up to 40 μg/mL liposome concentration, highlighting the excellent biocompatibility of ImmuLip. It is important that the concentration of ImmuLipCP for following in vivo study was below the cytotoxicity window shown in Figure 7A. As both CPT-11 and panobinostat were reported to exhibit a cancer cell killing effect by causing cell apoptosis [46,47], we test the cytotoxicity of drugs delivered through liposomes using flow cytometry for cell apoptosis assays. The U87DR cells treated with LipCP or ImmuLipCP were labelled with Annexin V/PI and represented separately in Q1, Q2, Q3 and Q4 quadrants as necrotic, late apoptotic, viable and early apoptotic cells (Figure 7B). The cell death mechanism is confirmed to be mainly due to late apoptosis, with the total apoptosis rate from targeted (ImmuLipCP) or non-targeted (LipCP) liposomes being at 45.5% and 29.2%, respectively. Undoubtedly, the mechanism responsible for the observed elevated apoptosis rate is associated with the pronounced increase of intracellular uptake of ImmuLip and the improved intracellular accumulation of drugs (Figure 5). Overall, the enhanced cancer cell killing effect originated from Ab-mediated targeted co-delivery of CPT-11 and panobinostat to GD-2 overexpressed cancer cells suggests the use of ImmuLipCP for in vivo anti-cancer therapy. 

#### 2.3.4. In Vitro Hemocompatibility

**Figure 8:**
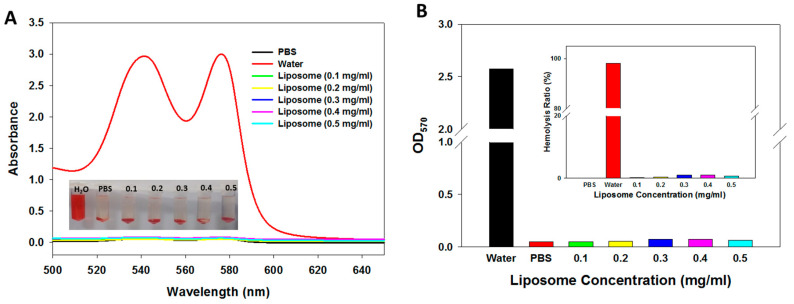
(**A**) Haemocompatibility of ImmuLip determined from the hemolysis assay after incubating ImmuLip with diluted red blood cells in phosphate buffered saline (PBS) at 37 °C for 2 h. Water and PBS were used as the positive and the negative controls, respectively. The full-wavelength absorption spectra of the supernatant as well as gross views of the samples (insert) are shown. (**B**) The absorbance at 570 nm (OD_570_) were used to calculate the hemolysis ratio shown in the insert.

With the growing number of studies reporting the hemocompatibility of liposomal formulation for drug delivery, it is important to evaluate the impact of sytematic adminstration of immunoliposomes on blood components before animal experiments [48]. This was assessed by determing whether ImmuLip will cause any damage to red blood cells (RBCs) from in vitro haemocompatibility assays. After incubating diluted RBCs with ImmuLip prepared in PBS for 2 h at 37 °C, we collected the supernatant to evaluate its absorption spectra. In addition to liposomes, diluted RBCs were incubated with PBS and water, which were used as the negative and positive controls, respectively. From Figure 8A, it is clear that the absorption spectra of all liposome samples were similar to that in PBS, which confirms that ImmuLip will not cause any damage to RBCs. In contrast, the absorption spectra of the supernatent collected after incubating RBCs with water shows strong absorption peaks at 540 nm and 570 nm, corresponding to the release of oxyhemoglobin caused by busting of RBCs in water. The gross views of all samples shown in the insert of Figure 8A further confirms the absorption spectra observed. The results were further quantified in terms of hemolysis ratio, which is the ratio of the difference in OD_570_ (absorbance at 570 nm) between the sample and the negative control (PBS) divided by the difference between the positive control (water) and the negative control (PBS). As shown in Figure 8B, the hemolysis ratio obtained for ImmuLip up to 0.5 mg/mL were lower than 2%, indicating negligible hemolysis. However, RBCs incubated in water shows more than 90% hemolysis ratio. With the excellent biocompatibility and hemocompatibility, ImmuLip is ready to be used in the following animal study as a nanocarrier for CPT-11 and panobinostat to evaluate anti-cancer efficacy in vivo [49]. 

### 2.4. In Vitro Experiments

#### 2.4.1. Biodistribution and Tumor Targeting In Vivo

**Figure 9:**
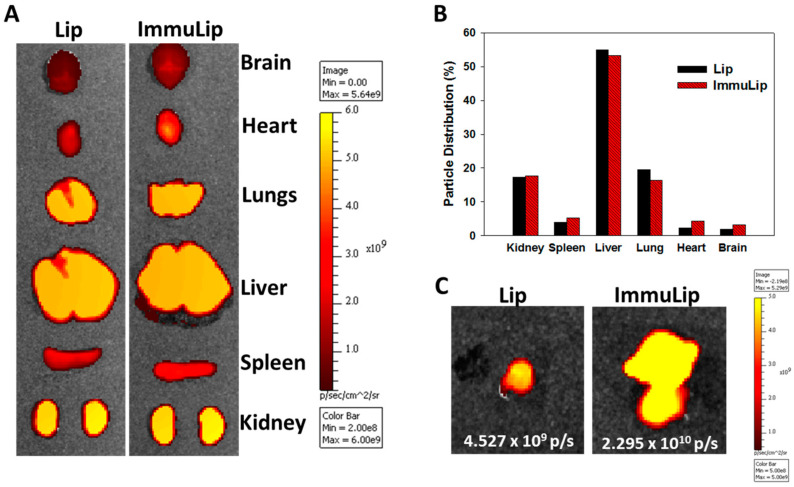
The biodistribution of Lip and ImmuLip in U87 tumor-bearing nude mice by injecting 100 μL solution containing Cy5.5 labelled liposomes (2 mg/mL) through the tail vein. The in vivo imaging system (IVIS) was used for ex vivo imaging of harvested organs after 4 h (**A**) and quantification of distribution of liposomes in each organ was calculated based on fluorescence intensity in each organ (**B**). The targeting effects of ImmuLip vs Lip was shown from the ex vivo IVIS imaging of explanted tumors (**C**).

With in vitro studies, we have illustrated the importance of anti-GD2 Ab density on targeting efficiency of ImmuLip to U87DR cell line, as mediated by the overexpressed GD2 on cell surface. To confirm the targeting effects in vivo, ImmuLip was labelled with Cy5.5 and injected intravenously (via the tail vein) into nude mice bearing high GD2-expression U87DR tumor by subcutaneously implanting U87DR cells in the right flank. Four hours after administration of Cy5.5-labelled ImmuLip (100 µg dose), the animal was subject to in vivo imaging system (IVIS) observation for biodistribution and tumor targeting. To conduct biodistribution assay, the mouse was sacrificed, followed by harvesting the organs to visualize the accumulation of liposomes in each organ by IVIS (Figure 9A). The distribution of Lip or ImmuLip among organs was calculated form the fluorescence intensity in each organ from the ex vivo images. As shown in Figure 9B, the particle is mainly accumulated in liver, kidney and lungs with no difference shown between Lip and ImmuLip. More than half of the administrated ImmuLip was accumulated in the liver as hepatic clearance represents the primary route of excretion for nanoparticles that do not undergo renal clearance [50]. The biodistribution study also suggests the in vivo stability of ImmuLip as more and more nanoparticles will be entrapped within the liver and spleen when the size of liposomes was increased beyond 150 nm [51]. The presence of fluorescence signal in kidney is reminiscent of appearance of liposomes with 50–100 nm size in diluted serum within 4 h (Figure 2A), as nanoparticles with size within that range were reported to be filtered out by the kidney [50]. The effect of GD2 targeting is demonstrated in the ex vivo tumor images in Figure 9C, which show 5.1 times higher fluorescence intensity of Cy5.5-labelled ImmuLip than Lip in U87DR tumors. The in vivo tumor targeting study thus matches the drastically enhanced intracellular uptake efficiency shown from the confocal microscopy (Figure 4) and flow cytometry analysis (Figure 5A) in vitro. Taken together, ImmuLip will be an excellent candidate for drug delivery to GD2-overexpressed tumors. 

#### 2.4.2. In Vivo Anti-Tumor Efficacy

**Figure 10:**
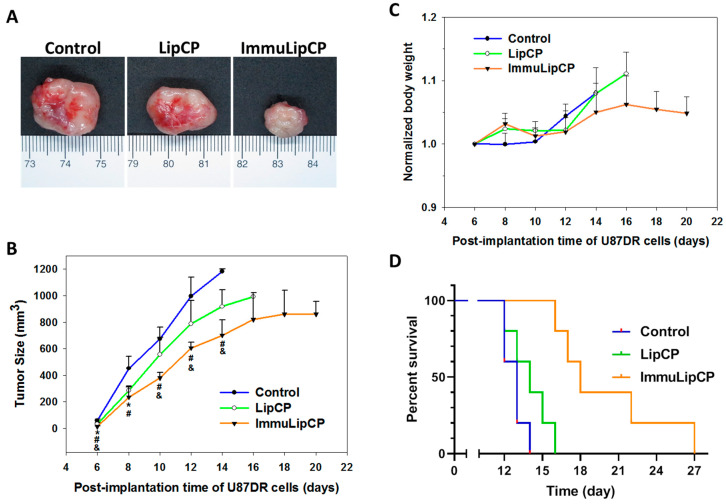
The in vivo anti-tumor efficacy was studied using a xenograft tumor model in nude mice by subcutaneous implantation of U87DR cells. The mice were divided into 3 groups and subject to intravenous injection of 100 μL of PBS (control), LipCP (7 mg/mL) or ImmuLipCP (7 mg/mL) on day 6, 8, 10 and 12. (**A**) The gross view of tumors retrieved on day 14. (**B**) The efficacy of treatment was evaluated from change of tumor size with time (*n* = 5). * *p* < 0.05, LipCP vs control; ^#^ *p* < 0.05 ImmuLipCP vs control; ^&^ *p* < 0.05 ImmuLipCP vs LipCP. (**C**) The change of body weight with time (*n* = 5). (**D**) The survival curve as obtained from the survival time of mice by sacrificing the animal when the tumor volume exceeds 1000 mm^3^ (*n* = 5).

To determine in vivo anti-tumor efficacy, we used a xenograft tumor model created in nude mice with subcutaneously implanted U87DR cells. Six days after tumor cell implantation, the mice were divided into three groups and treated by intravenous injection of PBS (control), LipCP and ImmuLipCP. Additional treatments were conducted on day 8, 10 and 12. The tumor size was monitored every two days and tumor volume was calculated as length × width × width ÷ 2. The mouse was sacrificed when the tumor volume exceeds 1000 mm^3^. The representative gross view image of explanted tumors on day 14 is shown in Figure 10A. It is clear from the image that tumors treated with ImmuLipCP demonstrated significant reduction in tumor size compared with other groups. The tumor growth curve in the xenograft model is shown in Figure 10B. The tumor volume of mice in the control group increased rapidly; followed by the group treated with LipCP. In contrast, the ImmuLipCP group showed significantly slower increase of tumor size. Indeed, due to the presence of targeting ligand, ImmuLipCP will be accumulated in the tumor more than LipCP (Figure 9B), which could lead to higher anti-tumor efficacy manifest from the slowest increase of tumor volume. Hence, passive targeting through the enhanced permeability and retention phenomenon with the nano-sized liposomes could be augmented by anti-GD2 Ab directed active targeting to improve the completeness of tumor destruction [52].

In addition to tumor volume measurements, we measured mouse body weight to assess chemotherapy-induced weight loss. The change of body weight was normalized to that on day 6 when the treatment starts and presented in Figure 10C. No significant changes in body weight were noted among all groups, indicating no adverse effect using dual drug-loaded liposomes. A survival curve of mice was constructed by setting 1000 mm^3^ tumor volume as the time for sacrificing the animal (Figure 10D). The mice in ImmuLipCP group showed prolonged median survival time (18 days) over mice in LipCP (14 days) and control (13 days) groups (Table 2). The survival time in the ImmuLipCP group is also significantly higher than that in LipCP and control groups (Table 2). Overall, the trend observed from mice survival is consistent with that from tumor volume and endorse the use of ImmuLipCP for cancer therapy in xenograft tumor model created with GD2 overexpressed U87DR cells. 

#### 2.4.3. Histological Analysis

**Figure 11:**
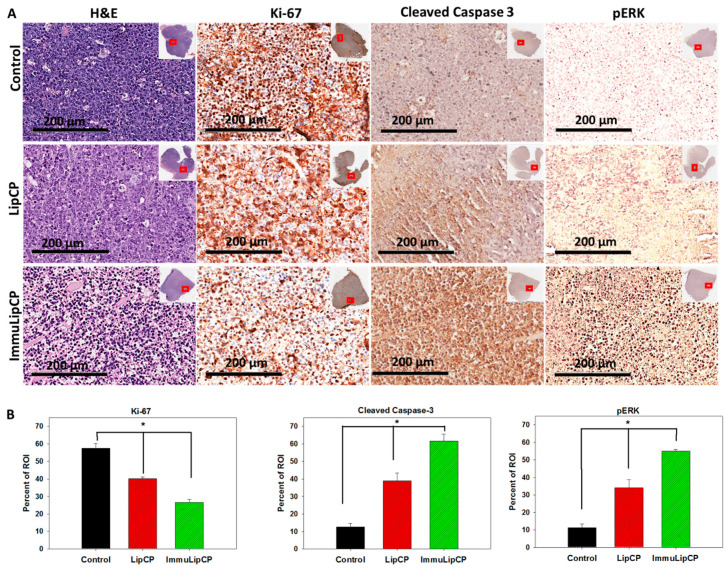
(**A**) The H&E and immunohistochemical (IHC) staining of Ki-67, cleaved caspase 3 and pERK after treatment with PBS (control), LipCP and ImmunLipCP (bar = 200 μm). The image was an enlarged view of the red square shown in the insert for each tumor section. (**B**) The quantitative analysis of IHC staining results (*n* = 5).

The therapeutic efficiencies of different treatments were further evaluated with a histological analysis of tumor sections from samples retrieved on day 14 (Figure 11A). The H&E staining of tumor tissue will contain irregular voids with purple outlines corresponding to the cells. This could be observed from the sample collected from the control group, which exhibits relatively higher cell intensity than the groups treated with drug-loaded liposomes. The ImmuLipCP treatment also shows much lower cell density than LipCP, corresponding to an increase in cell death with treatment. This was further confirmed from the immunohistochemical (IHC) staining of the cell proliferation marker Ki-67. The IHC images of tissue stained with Ki-67 antibody confirms the presence of actively proliferating cells in the control group, similar to the result obtained from H&E staining. However, when tumor bearing mice were subject to LipCP treatment, proliferation of tumor cell was arrested to result in less Ki-67 immunoreactivity than the control group due to apoptosis of U87DR cells. Most importantly, the tumor treated with ImmuLipCP shows the weakest immunoreactivity of Ki-67 and the most effective inhibition of cancer cell proliferation. As less cell proliferation maker was expressed in tumors treated with ImmuLipCP, more cellular apoptosis could be suggested to be induced through targeted delivery of CPT-11 and panobinostat.

The trend seen in both H&E and Ki-67 staining was further supported by staining the apoptosis protein marker, cleaved caspase 3 and phospho-ERK (pERK), in the tumor tissue, which were reported to be reliable indicators of cell apoptosis caused by CPT-11 [43]. The activation of caspase 3 is a key event in cell apoptosis and a reliable indicator of apoptosis [53]. When cancer cells are treated with chemotherapeutics, activation of stress-related signaling pathways is an important outcome at the cellular level and a signaling pathway involving the pERK protein could lead to endoplasmic reticular stress-induced apoptosis [54]. As shown from Figure 11A, we could observe the most pronounced expression of cleaved caspase 3 and pERK protein in the tumor tissue section of ImmuLipCP-treated mice (Figure 10A). Moreover, the trend was similar for the other cell apoptosis marker pERK. The IHC staining of apoptosis marker proteins in tumors from in vivo study thus is consistent with the in vitro apoptosis result obtained from the flow cytometry analysis. 

The qualitative results for the IHC analysis were further quantified by Pax-It image analysis software from 5 different areas in the enlarged histological image. As shown in Figure 11B, the positively stained areas, shown as percent of region of interest (ROI) for Ki-67 is 57.4%, 40.2% and 26.6%, respectively, for mice treated with PBS, LipCP and ImmuLipCP. The less than half Ki-67-positive area identified from the histological image of the ImmuLip group compared with the control strongly supports the use of this DDS in arresting the cell proliferation of tumor cells. In contrast, the results obtained for both apoptosis marker proteins, cleaved caspase 3 and pERK, clearly show a reverse order as expected. Compared to the control group, the percent of ROI corresponding to cleaved caspase 3 (or pERK) of ImmuLipCP were ~5 times that of control and 3.2 (or 1.6) times that of LipCP, due to a significantly higher cell apoptosis rate.

#### 2.4.4. Hematological Analysis

The in vivo toxicities of the treatment groups were tested from hematological and biochemistry analysis of blood samples collected from sacrificed mice (*n* = 5). As shown in Table 3, the administration of LipCP or ImmuLipCP did not significantly alter the level of blood counts and hepatic or renal functions from hematologic study when compared with the control group (*p* > 0.05). The non-significant change of blood urea nitrogen (BUN) and creatinine level indicates that treatment with drug-loaded liposomes causes no significant toxicity towards mice to influence kidney functions. Similarly, the non-toxicity shown from the liver functions was confirmed from the unaltered values of aspartate transaminase (AST), alanine transaminase (ALT). Thereby, as in body weight change shown in Figure 10C, co-delivery of CPT-11 and panobinostat by LipCP or ImmuLipCP will not lead to significant adverse effects from hematological analysis, which has been a prime concern of free drug delivery. 

## 3. Materials and Methods

### 3.1. Materials

1,2-Distearoyl-sn-glycero-3-phosphocholine (DSPC) was procured from Avanti Polar Lipids (Alabaster, AL, USA). Dimethyldioctadecylammonium bromide (DDAB), cholesterol and Triton X-100 were obtained from Sigma-Aldrich (St. Louis, MO, USA). N-(aminopropyl polyethyleneglycol)carbamyl-distearoylphosphatidyl-ethanolamine with 5000 PEG chain molecular weight (DSPE-PEG-NH_2_) was purchased from NOF Co. (Tokyo, Japan). 5(6)-Carboxyfluorescein N-hydroxysuccinimide ester (fluorescein NHS) was acquired from Nanocs Inc. (New York, NY, USA) while cyanine 5.5-NHS (Cy5.5-NHS) was from Lumiprobe Co. (Hunt Valley, MD, USA). Panobinostat and CPT-11(irinotecan hydrochloride salt) were purchased from AdooQ BioScience (Irving, CA, USA) and LC Laboratories (Woburn, MA, USA), respectively. The U87MG human glioblastoma cell line (ATCC HTB1) was obtained from the American Type Culture Collection (Manassas, VA, USA). The anti-GD2 Ab (14G2a) was isolated and purified from hybridoma cell culture supernatant using a protein G column (HiTrap Protein G HP, Cytiva, Marlborough, MA, USA). 

### 3.2. Preparation of Liposomes (Lip)

The liposomes were prepared by the thin film hydration method in phosphate buffered saline (PBS), as described in our previous work [10]. In brief, DSPC, cholesterol, DDAB and DSPE-PEG-NH_2_ with 6:4:1:0.5 molar ratios were dissolved in a 100 mL round-bottom flask containing 3 mL of chloroform/methanol (2:1(v/v)) mixture to prepare a 10 mM lipid solution. The lipid solution was converted into a lipid film using a roto-evaporator (EYELA N- 1200AVF, Tokyo, Japan) working at 100 psi and 55 °C. After 20 min evaporation, the film formed on the wall of the round-bottom flask was further dried overnight in a vacuum oven, for complete removal of trace organic solvents. Subsequently, the lipid film was hydrated for 20 min at 55 °C with 10 mL PBS in the round-bottom flask and the resultant liposome suspension was sonicated for 20 min using a Q700 ultrasonicator (Qsonica, Newtown, CT, USA) maintained at 30% amplitude and 5 s pulse on/off cycles. By extrusion of liposomes through a polycarbonate filter of 220 nm pore size in an Avanti^®^ Mini Extruder (Avanti Polar Lipids, Inc., Alabaster, AL, USA), we produced small unilamellar vesicles (SUVs) or large unilamellar vesicles (LUVs) from multilamellar vesicles (MLVs). Compared to the MLVs, the SUVs and LUVs are more stable and could achieve higher drug encapsulation efficiency. 

### 3.3. Preparation of Drug-Loaded Liposomes (LipCP)

Various approaches, including active and passive methods, were used to develop drug-loaded liposomal formulations [55]. As the freeze-thaw method has been shown to increase drug encapsulation capacity in liposomes by decreasing the lamellarity of formed liposomal particles, we adopted this method for the co-encapsulation of CPT-11 and panobinostat in this study [56,57]. This method is an active method for drug loading by alternatively exposing the liposome sample to different temperatures in high gradients. For this purpose, the liposomal solution prepared above in PBS was mixed with a drug solution containing panobinostat (2 mg/mL) and/or CPT-11 (0.5 mg/mL). The mixture was incubated in a 60 °C water bath for 30 min, followed by freezing in a −20 °C refrigerator for 1 h and thawing again at 60 °C for 30 min. This freeze-thaw cycle was repeated five times and finally the solution was centrifuged at 35,000× *g* for 30 min to remove unencapsulated drugs in the supernatant. To determine the drug encapsulation efficiency (EE), drug-loaded liposomes were treated with a lysis buffer (0.1% Triton X-100 in PBS, pH 7.4) at 37 °C for 30 min. The solution was centrifuged at 35,000× *g* for 30 min and the supernatant was collected for analysis of drug concentration by high performance liquid chromatography (HPLC). A Thermo Finnigan Surveyor HPLC System was used, with a Hypersil™ ODS C18 column (5 µm) and 1:1(v/v) 0.1% orthophosphoric acid/acetonitrile as the mobile phase and operating at 0.3 mL/min flow rate. The concentration of CPT-11 and panobinostat were detected with an UV detector at 370 nm and 270 nm, respectively. The drug encapsulation efficiency (%) is weight of encapsulated drug ÷ weight of drug initially added × 100 (*n* = 5). 

### 3.4. Preparation of Immunoliposomes (ImmuLip) and Drug-Loaded Immunoliposomes (ImmuLipCP)

The immunoliposomes with (ImmuLipCP) or without (ImmuLip) the drug were fabricated by conjugating anti-GD2 Ab to LipCP or Lip following procedures reported before with some modifications [38,58]. We used the -NH_2_ functional group on liposome surface originated from DSPE-PEG-NH_2_ in the lipid bilayer, for conjugating with the -CHO groups in anti-GD2 Ab by spontaneous covalent bond formation (Figure 1A). To activate anti-GD_2_ Ab for reactivity towards the -NH_2_ group, sodium periodate (NaIO_4_) solution (0.1 M) was added dropwise into an anti-GD2 Ab solution and stirred for 20 min in dark. During this step, the sugar-based anti-GD_2_ Ab undergoes ring opening to produce reactive aldehyde groups (Figure 1A). The activated anti-GD_2_ Ab was purified by passing through a PV-10 desalting column and diluted with 0.2 M sodium carbonate buffer (pH 9.5) to 40 μL/mL. Five hundred microliter of Lip or ImmuLip solution (5 mg/mL) was added slowly into the above mixture and allowed to react for 5 min to conjugate anti-GD_2_ Ab to liposome surface by forming covalent bonds. Subsequently, the covalent bonds were stabilized by adding NaBH_4_ to reach 4 mg/mL final concentration and incubated at 4 °C for 1 h. The liposomes were recovered by centrifugation at 40,000× *g* for 30 min and the concentration of unconjugated Ab in the supernatant was analyzed quantitatively using Pierce™ BCA protein assay kit (Thermo Fisher Scientific, Waltham, MA, USA) using a calibration curve constructed with the anti-GD2 Ab. 

### 3.5. Physico-Chemical Characterization of Liposomes and Drug Release

The Lip and ImmuLip were characterized for morphology using a cryo-transmission electron microscope (Cryo-TEM). To prepare the cryo-TEM sample, 2 µL of liposome suspension (100 µg/mL) was dropped on a holey carbon grid that has been treated by glow discharge [59]. Following incubation for 20 s, the grid was blotted quickly and plunged in precooled liquid ethane. The grid was transferred into a cryo-TEM holder in presence of liquid nitrogen and visualized under a JEOL JEM 2100 Plus Cryo-TEM. The particle size from dynamic light scattering (DLS) and the zeta potential were analyzed by Zetasizer (Nano ZS 90, Malvern, UK) using a 50 µg/mL liposome suspension in PBS (pH 7.4) (*n* = 3). To determine the in vitro stability of liposomes mimicking the in vivo condition, a 5 µg/mL ImmuLipCP solution was prepared in 10% FBS/90% PBS and filtered through a 220 nm filter. After incubating at 37 °C for predetermined times, the solution was analyzed by nanoparticle tracking analysis (NTA) using NanoSight LM10 (Malvern Panalytical, Malvern, UK) equipped with a 405 nm laser. Based on Brownian motion, the scattered light intensity from the particle upon laser exposure could determine the concentration and diameter of liposomes [60,61]. 

The drug release profile from liposomes was determined in 1 mL PBS (pH 7.4) in a shaking water bath at 37 °C. After shaken at 100 rpm for predetermined times, the solution was centrifuged at 30000× *g* for 30 min and the supernatant was collected for analysis of drug concentration by HPLC as described before. Fresh PBS was added to replenish the volume of removed supernatant and the drug release experiment was continued. The cumulative drug release (%) is cumulative weight of drug released form liposomes ÷ weight of drug encapsulated in the liposomes × 100 (*n* = 5).

### 3.6. In Vitro Experiments

#### 3.6.1. Establishing Drug-Resistant Glioma Cell Line (U87DR) with High GD2 Expression

Most of the tumor cells are characterized by the abnormal cell proliferation resulting from the cyclin-dependent kinases (CDKs) activation [62]. Preventing tumor cell division and proliferation by blocking CDK4 and CDK6 with the help of a CDK4/6 inhibitor like palbociclib is a potential way for cancer therapy. However, palbociclib-resistant cell line was reported to hamper this approach [63]. To develop a high GD2 expression glioma cell line (U87DR), we adopted methods reported before for establishing palbociclib-resistant estrogen receptor positive breast cancer cells and carried out a long-term culture of U87MG cells in the presence of palbociclib [64,65]. In brief, the U87MG cells were cultured in the Dulbecco’s Modified Eagle’s Medium-High Glucose (DMEM-high glucose) containing 10% fetal bovine serum (FBS) and 0.2 µM of palbociclib. The medium was changed every 3 days. After observing cell growth, an increasing gradient of palbociclib concentration up to 1 μM was used for culture to make develop drug resistant U87MG variant. The palbociclib-resistant U87DR cell line could be established this way within 3 months, which was maintained in 1 μM palbociclib. The morphological changes of cells were monitored to confirm the conversion of U87MG to U87DR and flow cytometry analysis was performed to determine the difference of GD2 expression between those cell lines. 

#### 3.6.2. GD2 Expression on U87MG and U87DR Cell Surface by Flow Cytometry

The expression of GD2 antigen on U87MG and U87DR surface was analyzed by flow cytometry analysis using anti-GD2 Ab (14G2a). To perform this assay, cells were trypsinized and detached from the culture plates. The cells were collected and washed with PBS for incubation with unlabeled anti-GD2 Ab (1 μg per 10^6^ cells) for 1 h. Followed by this, the cells were washed in PBS containing 1%FBS and 0.02% sodium azide. Subsequently, the cells were incubated with fluorescein isothiocyanate (FITC)-labeled anti-mouse IgG (1:1000) for 40 min and washed twice in PBS. The samples were then analyzed using FACS instrument (SONY SA3800 spectral cell analyzer) where 10,000 events were collected for each sample. The data so obtained were further analyzed using the FlowJo software to determine the extent of GD2 expression. 

#### 3.6.3. Intracellular Particle and Drug Uptake

The intracellular uptake of Lip and ImmuLip were determined from confocal microscopy and flow cytometry analysis, for analyzing cell trafficking and targeting efficiency mediated by anti-GD2 Ab. To perform the qualitative analysis, U87DR cells were seeded on a 15 mm coverslip in a 24-well culture plate at 5 × 10^4^ cells per well and cultured in cell culture medium (DMEM supplemented with 10% FBS) for 24 h in a humidified CO_2_ incubator at 37 °C under 5% CO_2_. After washing with PBS, the cells were incubated with green fluoresce-producing liposomes (50 μg/mL in cell culture medium) that were prepared by labelling liposomes with fluorescein NHS through reaction between -NH_2_ groups of liposomes and -NHS group of fluorescein NHS. After 24 h incubation, the cells were washed with PBS and fixed with 4% paraformaldehyde. Subsequently, the cells were further treated with Triton X-100 (0.1% in PBS) for 10 min at room temperature and the cell cytoskeleton was stained with red fluorescence-producing phalloidin-tetramethylrhodamine B isothiocyanate (1%, Sigma-Aldrich, St. Louis, MO, USA). In the next step, the cell nuclei were visualized by counterstaining with blue fluorescence-producing Hoechst 33342 (1 μg/mL, Thermo Fisher Scientific). The intracellular uptake of liposomes was observed under a confocal laser scanning microscope (Zeiss LSM 510 Meta) at excitation/emission wavelength of 350 nm/451 nm for blue, 492 nm/517 nm for green and 577 nm/590 nm for red fluorescence signal. All three fluorescence were visualized individually at its corresponding wavelengths and then merged together to visualize the cellular internalization of liposomes. To study the effect of anti-GD2 Ab density on targeting efficiency, we also used immunoliposome (ImmuLip-L) prepared with 20% of the anti-GD2 Ab concentration used for preparing ImmuLip and observe its intracellular uptake by confocal microscopy.

The intracellular uptake of Lip (liposomes without anti-GD2 Ab), ImmuLip-L (liposome with low anti-GD2 Ab) and ImmuLip (liposome with high anti-GD2 Ab) was also determined quantitatively by flow cytometry analysis using fluorescein-labelled liposomes. To perform this analysis, U87DR cells were seeded in T-25 tissue culture flasks with a cell density of 5 × 10^5^ cells per flask and incubated at 37 °C under 5% CO_2_ atmosphere. Subsequently, the cells were treated separately with Lip, ImmuLip-L or ImmuLip at 50 μg/mL final liposome concentration for 24 h. After washing with PBS, the cells were trypsinized and centrifuged at 2000× *g* for 5 min for collecting the cell pellet. The cell pellet was re-suspended in 500 μL binding buffer, followed by determining the fluorescence intensity corresponding to intracellularly accumulated liposome using a FACS instrument (Attune NxT Flow cytometer, Life Technologies) with a 530 nm emission filter.

To confirm the carrier-dependent accumulation of drugs, the intracellular drug uptake efficiency was also analyzed by flow cytometry analysis through the blue fluorescence intensity from CPT-11. To perform this experiment, cells in a T-25 cell culture flask (5 × 10^5^ cells per flask) were treated with LipCP, ImmuLipCP or free CPT-11/panobinostat at the same drug dosage (20 μg/mL CPT-11). After 24 h incubation, the cell pellet was collected and analyzed for fluorescence intensity of CPT-11 at 405/450 nm using the same protocol established for liposome uptake [6]. The flow cytometry analysis of particle and drug uptake was duplicated to confirm reproducibility of the data and representative images are shown.

#### 3.6.4. In Vitro Cytotoxicity

The cell viability of U87DR cells after different treatments in vitro was determined using the MTS cell proliferation assay from the solution absorbance of metabolites of 3-(4,5-dimethylthiazol-2-yl)-5-(3-carboxymethoxyphenyl)-2-(4-sulfophenyl)-2H-tetrazolium (MTS) salt [66]. The biocompatibility of ImmuLip was determined by seeding U87DR cells in 96-well cell culture plate at 2.5 × 10^3^ cells/well and incubated for 12 h. The cells were separately treated with different concentrations of ImmuLip (up to 80 μg/mL) for 24 h or 48 h at 37 °C. Subsequently, the cell culture medium was replaced with 100 μL PBS and 20 μL MTS reagent for incubation at 37 °C for 3 h. The solution absorbance was analyzed using an enzyme-linked immunosorbent assay (ELISA) plate reader (BioTek Synergy HT) at 490 nm wavelength to detect the product produced from reduction of MTS reagent through the mitochondria activity of live cells. The analysis was performed five times for each group and the average values obtained were then normalized to those obtained for the control group using cell culture medium alone, which was taken as 100% (*n* = 5).

The in vitro cytotoxicity of CPT-11 and/or panobinostat was evaluated in a similar way using U87DR cells to determine the synergistic effect of drug combination. The cells were seeded in 96- well culture plate and incubated with different concentrations of CPT-11 (1 μM to 40 μM), panobinostat (10 nM to 80 nM) or combinations of CPT-11 and panobinostat (with fixed panobinostat concentration or fixed CPT-11 concentration). After 24, 48 and 72 h incubation, the relative cell viability was determined by the MTS assay as before (*n* = 5). The cell viability obtained for single drug and dual drug was analyzed using the CompuSyn software (ComboSyn Inc., Paramus, NJ, USA) for the evaluation of the combined effects of CPT-11 and panobinostat [67].

The cytotoxicity of drug-loaded liposomes (LipCP and ImmuLipCP) was determined from flow cytometry analysis of the apoptosis rates of U87DR cells using an apoptosis detection kit (Thermo Fischer Scientific) that contains fluorescein isothiocyanate-labeled Annexin V (Annexin V-FITC) and propidium iodide (PI). In brief, the cells were seeded in T-25 culture flask at a cell density of 5 × 10^5^ cells/flask and incubated with LipCP and ImmuLipCP at 5 nM panobinostat and 2 μM CPT-11 dosages for 24 h. The cells were trypsinized and centrifuged at 2000× *g* for 5 min. The collected cell pellet was incubated with Annexin V-FITC for 30 min to label the live cells and early apoptotic cells. After labelling the late apoptotic and necrotic cells with PI, cells were analyzed for the fluorescence emission at 530 nm/570 nm using a FACS instrument (Attune NxT Flow cytometer, Life Technologies, USA). The flow cytometry analysis of cytotoxicity was duplicated to confirm the reproducibility and representative images are shown.

#### 3.6.5. In Vitro Hemocompatibility

The blood compatibility of was analyzed by evaluating possible hemolysis induced by ImmuLip [68]. Briefly, whole blood sample collected from rats was diluted ten times with PBS and 0.3 mL diluted blood sample was mixed with 1.2 mL test sample and incubated at 37 °C for 2 h. The diluted blood sample treated with deionized water and PBS were used as the positive and negative controls, respectively. The mixture was centrifuged and the absorbance of the supernatant was recorded at 540 nm (OD_540_) using a UV-Vis spectrophotometer. The hemolysis ratio was calculated by dividing the difference in OD_540_ between the sample and the negative control by the difference in OD_540_ between the positive control and the negative control.

### 3.7. In Vivo Experiments

All in vivo experiments were performed as per protocols that have been approved by the Chang Gung University’s Institutional Animal Care and Use Committee (IACUC Approval No.: CGU108-214). The biodistribution and in vivo targeting efficiency of ImmuLip and Lip was performed in nude mice bearing U87DR tumor by subcutaneous injection of 10^5^ U87DR cells to the right flank of 6–8 week old mouse. To perform the biodistribution study, Lip and ImmuLip were labelled with Cy5.5 using Cy5.5-NHS. One hundred microliter of Cy5.5-labelled liposomes (1.92 μg/mL) was administered intravenously via tail vein. Four hours after administration, the mice were sacrificed and organs including kidney, spleen, liver, lungs, heart and brain were harvested to visualize the accumulation of particle in each organ with an in vivo imaging system (IVIS) (Xenogen IVIS 200, Caliper Life Sciences, Hopkinton, MA, USA). The tumor tissue was explanted from each mouse and analyzed by IVIS to compare the accumulation of Lip and ImmuLip in vivo for targeting efficiency.

The in vivo antitumor efficacy was studied in U87DR tumor bearing mice prepared as before. Six days after U87DR cell implantation, the mice were randomly divided into 3 groups and subject to intravenous injection of 100 μL of PBS (control), 100 μL of 7 mg/mL LipCP or 100 μL of 7 mg/mL ImmuLipCP at 12 mg/kg panobinostat and 7 mg/kg CPT-11 drug dose (*n* = 5, each group). All mice were treated similarly again on day 8, 10 or 12. The tumor size and the mouse body weight were recorded. The tumor was explanted from one mouse on day 14 for gross observation. The tumor size was measured using a caliper and tumor volume was calculated from length × width × width ÷ 2. Mice were sacrificed when the tumor volume exceeded 1000 mm^3^, from which survival time of the animal was determined. 

For histology, tumor tissues were collected after euthanizing the mice and fixed in 12% formaldehyde. The tissues were sectioned into 5-μm thickness on glass slide after paraffin-embedment. Samples were then subject to hematoxylin and eosin (H&E) staining following standard protocols. Another set of slides were allowed to undergo immunohistochemical (IHC) staining using rabbit primary antibody for Ki-67 (Cell Signaling anti-Ki-67, 1:400), pERK (Cell Signaling anti-phospho-p44/42 MAPK, 1:400) and cleaved caspase-3 (Cell Signaling anti-cleaved caspase-3, 1:600). After incubating with anti-rabbit secondary antibody (ImmPRESS^®^ HRP universal antibody) for color development counterstained with hematoxylin for nucleus, images were taken under Aperio ScanScope^®^ XT and analyzed by the ImageScope software. The immunoreactivity in captured images was then quantified in a 70 μm × 150 μm measuring frame by using the PAX-it image analysis software. The protein expression from the level of immunoreactivity was expressed as the percentage of the area in the region of interest (ROI) of the measuring frame that contained immunoreactivity. Five different areas form an image were selected for the quantification (*n* = 5). 

To evaluate systemic toxicity, blood samples from sacrificed mice (*n* = 5) were collected in BD Microtainer^®^ blood collection tubes with K2EDTA (Becton, Dickinson and Company, Franklin Lakes, NJ, USA). The collected blood was subject to hematologic analysis of white blood cell (WBC), red blood cell (RBC), platelet (PLT), hemoglobin (HGB) and hematocrit (HCT), as well as biochemical analysis of major organ functions from aspartate transaminase (AST), alanine transaminase (ALT), blood urea nitrogen (BUN) and creatinine (CRE). 

### 3.8. Statistical Analysis

All quantitative results were expressed as mean ± standard deviation (SD). To compare means of different groups, the one-way analysis of variance (ANOVA) analysis with Tukey’s HSD post-hoc test was used for statistical analysis. Differences were considered to be significant at *p* < 0.05. 

## 4. Conclusions

In this study, we demonstrated active targeting of GD2 overexpressed U87DR glioma cells by anti-GD2 Ab conjugated liposomes (ImmuLip). By finding the drug concentrations for synergism interaction, we successfully used this nanostructured vehicle for co-delivery of CPT-11 and panobinostat (ImmuLipCP) for targeted synergistic combination chemotherapy. Due to the overexpression of GD2 on U87DR cell line, ImmuLipCP results in active tumor targeting and effective chemotherapy. The in vitro experiments confirm the effective intracellular uptake of ImmuLip by U87DR, which shows 6-fold increase of uptake efficiency over non-targeted Lip and dependent on conjugated Ab density on liposome surface. Endowed with efficient active targeting ability, ImmuLipCP provides impressive enhancement of intracellular drug concentration to provide enhanced in vitro cytotoxicity by inducing cell apoptosis with synergistic action of CPT-11 and panobinostat. Moreover, the biocompatibility and hemocompatibility test ImmuLip supports its use for in vivo experiments. In xenograft model with U87DR tumor bearing nude mice, the 5.1-fold higher accumulation rate of targeted liposomes over non-targeted ones supports the in vitro targeting efficacy for drug delivery. The tumor bearing mice treated with ImmuLipCP showed similar body weight change and hematologic analysis results with the control and LipCP group, suggesting a highly compatible tumor treatment modality. Most importantly, mice treated with ImmuLipCP showed significantly reduced tumor growth rate and prolonged survival time. Overall, the results confirm that ImmuLipCP was an effective GD2-targeted cancer treatment modality to significantly enhance the anti-cancer therapeutic efficacy in U87DR tumors. The Ab-mediated liposome-based drug delivery system could provide a paradigm for targeted delivery of chemotherapeutics into tumor cells.

## Figures and Tables

**Table 1:** Size and zeta potentials values (*n* = 3, mean ± SD).

Samples ^1^	Size From DLS (nm) ^2^	PDI ^3^	Zeta Potential (mV)
Lip	130.9 ± 5.2	0.188 ± 0.03	14.7 ± 1.1
LipCP	147.5 ± 3.0	0.196 ± 0.02	13.3 ± 0.6
ImmuLip	168.4 ± 9.8 *	0.275 ± 0.02	9.6 ± 0.7
ImmuLipCP	160.3 ± 7.1 ^#^	0.081 ± 0.04	8.3 ± 1.0

^1^ Lip: liposomes, LipCP: liposomes loaded with CPT-11 and panobinostat, ImmuLip: immunoliposomes, ImmuLipCP: immunoliposomes loaded with CPT-11 and panobinostat. ^2^ The average particle diameter measured from dynamic light scattering (DLS). ^3^ PDI: polydispersity index. * *p* < 0.05 compared with Lip, ^#^ *p* < 0.05 compared with LipCP.

**Table 2:** Survival times for U87DR tumor-bearing mice after different treatments.

Group ^1^	Median Survival Time (days)	Survival Times (days) ^2^
Control	13	12.8 ± 0.7
LipCP	14	14.0 ± 1.4 *
ImmuLipCP	18	20.0 ± 4.0 *^,#^

^1^ Control: treated with PBS, LipCP: treated with liposomes loaded with CPT-11 and panobinostat, ImmuLipCP: treated with immunoliposomes loaded with CPT-11 and panobinostat. ^2^ Mean ± SD, *n* = 5. * *p* < 0.05 compared with control, ^#^ *p* < 0.05 compared with LipCP.

**Table 3:** Blood analysis for evaluation of systemic toxicity (*n* = 5, mean ± SD).

Analysis	Item ^1^	Unit	Control	LipCP	ImmuLipCP
Hematology	WBC	10^3^ cells/μL	3.2 ± 0.35	3.1 ± 1.62	3.2 ± 0.45
	RBC	10^6^ cells/μL	7.9 ± 0.33	7.3 ± 0.28	7.4 ± 0.25
	HGB	g/dL	12.5 ± 0.82	11.8 ± 0.83	11.7 ± 0.43
	HCT	%	37.6 ± 1.38	35.0 ± 1.17	32.7 ± 2.04
	PLT	10^3^ cells/μL	992 ± 174.9	1041.3 ± 140.5	1038.7 ± 277.8
Clinical Chemistry	AST	U/L	428.3 ± 75.9	440.3 ± 100	375 ± 184.6
	ALT	U/L	53.3 ± 13.9	55.7 ± 11.0	53.7 ± 20.7
	BUN	mg/dL	31.0 ± 1.73	33.7 ± 2.49	31.5 ± 2.49
	CREA	mg/dL	0.23 ± 0.06	0.27 ± 0.05	0.25 ± 0.01

^1^ WBC: white blood cell; RBC: red blood cell; HGB: hemoglobin; HCT: hematocrit; PLT: platelet; AST: aspartate transaminase; ALT: alanine transaminase; BUN: blood urea nitrogen; CREA: creatinine Values are means ± standard deviation (SD) of four independent measurements.

## Supplementary Materials

The following are available online at https://www.mdpi.com/2072-6694/12/11/3211/s1, Figure S1. The cell morphology and the expression levels of GD2 of U87MG and U87DR cell lines from flow cytometry analysis (bar = 100 μm).
