# Supplementary material for: Co-Delivery of CPT-11 and Panobinostat with Anti-GD2 Antibody Conjugated Immunoliposomes for Targeted Combination Chemotherapy
Source: Cancers (Basel). 2020 Oct 31;12(11):3211. doi: 10.3390/cancers12113211 (PMC7692704; doi:10.3390/cancers12113211)
Supplement: Supplementary file 1 [file cancers-12-03211-s001.pdf]

Supplementary Materials: Co-Delivery of CPT-11 and Panobinostat With Anti-GD2 Antibody Conjugated Immunoliposomes for Targeted Combination Chemotherapy

Gils Jose, Yu-Jen Lu, Jung-Tung Hung, Alice L. Yu and Jyh-Ping Chen

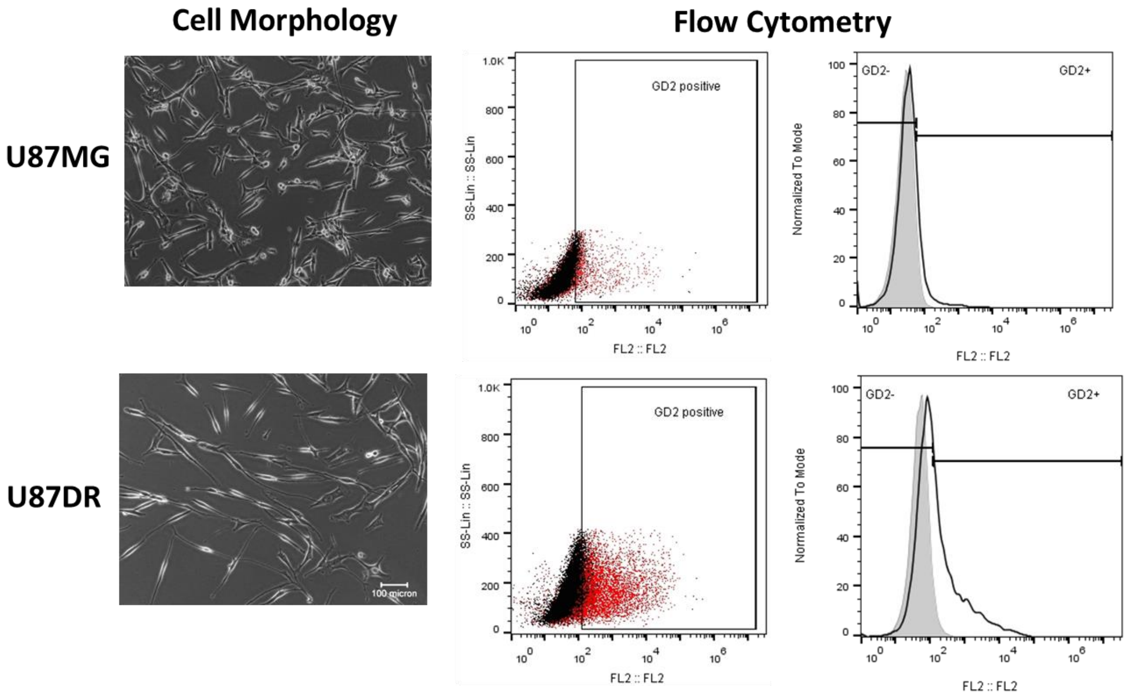

Figure S1. The cell morphology and the expression levels of GD2 of U87MG and U87DR cell lines from flow cytometry analysis (bar = 100 μm).

© 2020 by the authors. Licensee MDPI, Basel, Switzerland. This article is an open access article distributed under the terms and conditions of the Creative Commons Attribution (CC BY) license (http://creativecommons.org/licenses/by/4.0/).
